# Education, Lifestyle Risk Factors, and Treatment Choices and Multiple Sclerosis Progression

**DOI:** 10.1001/jamanetworkopen.2025.20142

**Published:** 2025-07-11

**Authors:** Jie Guo, Tomas Olsson, Jan Hillert, Lars Alfredsson, Anna Karin Hedström

**Affiliations:** 1 Department of Nutrition and Health, China Agricultural University, Beijing, China; 2 Department of Clinical Neuroscience, Karolinska Institutet, Stockholm, Sweden; 3 Institute of Environmental Medicine, Karolinska Institutet, Stockholm, Sweden; 4 Center for Occupational and Environmental Medicine, Region Stockholm, Stockholm, Sweden

## Abstract

**Importance:**

The implications of socioeconomic factors, including educational level, for multiple sclerosis (MS) progression remain unclear. Understanding whether educational level directly affects MS outcomes or is confounded by lifestyle risk factors and treatment choices could inform personalized care strategies.

**Objective:**

To investigate the association between educational level and outcomes related to MS, including worsening of disability, cognition, and health-related quality of life, after adjusting for potential confounding factors or mediation by lifestyle factors and treatment.

**Design, Setting, and Participants:**

This cohort study used data from 2 large, population-based case-control studies conducted in Sweden from April 2005 to December 2019 that used Swedish MS Registry data with detailed clinical and sociodemographic information. Patients with relapsing-onset MS aged 25 years or older at disease onset after 1995 were followed up from diagnosis until April 6, 2022, with a mean (SD) follow-up time of 10.4 (5.4) years. Data analysis was performed from July 2024 to November 2024.

**Exposure:**

Educational level categorized as presecondary (9 to 10 years of compulsory school), secondary (2 to 4 years of high school or college), and postsecondary (higher university education) July 2024 to November 2024.

**Main Outcomes and Measures:**

The primary outcome was confirmed disability worsening defined as a 1-point increase in Expanded Disability Status Scale (EDSS) score sustained across 2 follow-up visits at least 24 weeks apart. Secondary outcomes were worsening of health-related quality of life, measured by the MS Impact Scale (MSIS-29) physical and psychological subscale scores, and cognitive disability worsening, measured by a decrease in Symbol Digit Modalities Test (SDMT) score. Cox proportional hazards regression was used to evaluate associations between educational level and disability progression.

**Results:**

Of 3695 participants with MS, 2656 (71.9%) were female, with a mean (SD) age at diagnosis of 39.1 (9.1) years. Lower educational level was associated with older age at disease onset (mean [SD] age at onset: 42.2 [10.2] years for presecondary educational level vs 36.0 [8.4] years for postsecondary educational level), worse baseline clinical status (mean [SD] EDSS 2.7 [2.0] for presecondary education vs 1.7 [1.5] for postsecondary education), and lower likelihood of receiving second-line therapies (mean [SD] 164 [36.9%] for presecondary education vs 869 [54.1] for postsecondary education). In unadjusted analyses, lower educational level was associated with faster disability progression (ie, worsening), but this association was no longer significant after adjusting for treatment and lifestyle factors (adjusted hazard ratio [AHR], 1.14; 95% CI, 0.97-1.33). No associations were found between educational level and changes in MSIS-29 scores (AHR, 1.14 [95% CI, 0.90-1.44] for the MSIS-29 physical subscale and AHR, 1.00 [95% CI, 0.79-1.26] for the MSIS-29 psychological subscale) or SDMT performance over the 15-year follow-up (AHR, 1.05; 95% CI, 0.76-1.46). Mediation analysis revealed that treatment and lifestyle factors accounted for 79.9% of the observed association between education level and disability progression.

**Conclusions and Relevance:**

In this cohort study of participants with MS, observed differences in disability worsening by educational level were largely accounted for by lifestyle and treatment factors, suggesting that educational level itself may not be independently associated with MS progression.

## Key Points

**Question:**

Is educational level associated with multiple sclerosis (MS) progression after adjusting for lifestyle factors and treatment type?

**Findings:**

In this cohort study of 3695 participants with MS, a lower educational level (presecondary education) was associated with faster MS-related disability progression in unadjusted analyses, but no associations remained after adjusting for lifestyle factors and treatment.

**Meaning:**

This study found that confounding factors may play a role in associations between educational level and MS outcomes or could be mediated by lifestyle factors and treatment choices, emphasizing the need to address disparities in treatment and lifestyle interventions to optimize outcomes.

## Teaser

This cohort study evaluates the association between educational level and disability progression in patients with multiple sclerosis.

## Introduction

Multiple sclerosis (MS) is a chronic and often disabling neurological disorder characterized by a highly variable disease course. While genetic factors play a considerable role in the risk of developing MS,^1,2^ they may not fully explain the wide variation in disease progression observed among patients. Increasing evidence suggests that lifestyle factors, such as smoking and body mass index (BMI), have a substantial impact on the course of the disease, changing the rate of disability progression.^3,4,5^ Identifying modifiable factors that affect MS progression remains critical, particularly those that could inform interventions to improve patient outcomes.

Several recent studies have reported that socioeconomic factors, such as educational level, are inversely associated with disability progression in MS.^6,7,8^ However, interpreting these findings in causal terms requires careful consideration of potential confounding and mediating variables, including access to treatment and lifestyle choices, both of which are known to vary with socioeconomic status. Moreover, the mechanisms by which education might alter MS progression are likely complex and may involve multiple indirect pathways.

A recent study using mendelian randomization suggested that higher educational level offered a protective benefit against MS progression,^8^ further raising the possibility that education could be an important factor in long-term disability outcomes in patients with MS. In the present study, we aimed to reassess the association between educational level and disability worsening in MS by conducting a comprehensive analysis of a large cohort of patients with MS. We adjusted for a broad range of potential confounders and mediators, including treatment and lifestyle factors, to better isolate the direct and indirect associations between education and disability worsening due to disease progression. We also aimed to investigate the potential association of educational level with health-related quality of life and cognitive disability worsening.

## Methods

### Design and Study Population

This cohort study includes patients from 2 population-based case-control studies: the Epidemiologic Investigation of Multiple Sclerosis (EIMS) study and the Genes and Environment in Multiple Sclerosis (GEMS) study.^12^ Both studies included the Swedish general population aged 16 to 70 years. The EIMS study recruited incident cases of patients with MS from more than 40 hospital-based and private neurology clinics between April 2005 and December 2019 (n = 3567), while the GEMS study identified prevalent cases of patients with MS (distinct from those identified in EIMS study) from the national Swedish MS registry between November 2009 and November 2011 (n = 6148). All patients met the McDonald criteria for MS.^9,10^ Participants completed a standardized questionnaire at the time of recruitment. The EIMS and GEMS questionnaires on environmental exposures and lifestyle factors were highly similar, with most questions identically worded. The response rates among patients were 93% for the EIMS study and 82% for the GEMS study. Both studies received approval from the Regional Ethical Review Board at Karolinska Institute and were conducted in accordance with the ethical standards outlined in the 1964 Declaration of Helsinki^11^ and its later amendments which extended to the present study. Written consent was obtained from study participants. More details on study design and methods were published previously.^12^

Of 9715 patients in the 2 studies, we excluded those with disease onset before 1995 (n = 4002) when disease-modifying treatments were not yet available, those who were younger than 25 years at disease onset (n = 480) to ensure that most participants had the opportunity to complete higher education, those with progressive-onset MS (n = 357), and those who were not followed-up with the Expanded Disability Status Scale (EDSS) in the Swedish MS registry (n = 537), leaving 3695 patients in the current study. The EDSS is used to measure disability and MS progression. Scores range from 0 (no disability) to 10 (death).

### Definition of Exposures

Educational level was categorized as presecondary, secondary, and postsecondary levels. We used self-reported data on educational levels, which we validated against data on formal education retrieved from Statistics Sweden. The rationale for using self-reported educational level is that education obtained outside Sweden can be registered and classified in Swedish databases only if individuals actively apply for recognition of their qualifications. Although this recognition and registration system exists, not all individuals choose to register their foreign education, which may lead to an underrepresentation of highly educated individuals. The highest level of education at the time of diagnosis was included in our analysis.

### Outcome Measures

In accordance with recommendations by the Swedish MS association, detailed information is continuously and routinely registered in the national Swedish MS registry.^13^ This includes data on medical treatment, disease activity, physical functioning, mental health, and quality of life. To study change in disability over time, baseline was defined as the date of MS diagnosis.

The primary outcome, confirmed disability worsening (CDW), was defined as an increase in the EDSS score of at least 1 point from the first recorded EDSS score sustained across 2 follow-up visits no less than 24 weeks apart (1.5 points if the baseline EDSS score was 0 or 0.5 points if the baseline EDSS score was ≥5.5). Secondary outcomes included changes in health-related quality of life measured by the Multiple Sclerosis Impact Scale 29 (MSIS-29).^14^ Scores range from 0 to 100 points, with an increase of 7.5 points or more in the MSIS-29 physical and psychological subscales defined as clinically significant worsening from the patient’s perspective based on recommended thresholds for identifying meaningful changes in the physical and psychological impact of MS.^15^ Cognitive performance was assessed using the Symbol Digit Modalities Test (SDMT), with scores ranging from 0 to 110 (higher scores indicate better cognitive performance.^16^ Cognitive disability worsening was defined as an 8-point or greater decrease in the SDMT score, a threshold suggested to indicate meaningful cognitive decline.^17^

### Statistical Analysis

Categorical variables are presented as frequency and percentage, whereas continuous variables are presented as mean (SD). Time from diagnosis to 24-week CDW as well as physical and psychological worsening from the patient’s perspective and cognitive disability worsening were analyzed using multivariable Cox proportional hazards regression. Follow-up time was calculated as the time from the baseline until the onset of the events of interest, study dropout, death, or end of follow-up (April 6, 2022), whichever occurred first. The proportional hazards assumption was tested through the Schoenfeld residuals. No violations of proportionality were observed.

To complement the time-to-event analyses, we applied linear mixed-effect models to describe the longitudinal score trajectories of the EDSS, MSIS-29 physical and psychological subscales, and SDMT. Educational levels, time (ie, years after diagnosis), and their interaction were included in the model to test the differences in the aforementioned trajectories. Random effects included a random intercept for individuals and a random slope for time.

All analyses were adjusted for age at diagnosis, sex, disease duration (time between clinical onset and MS diagnosis), baseline EDSS score, receipt of disease-modifying therapy (none, only first-line therapy [interferon β-1a, interferon β-1b, glatiramer acetate, dimethyl fumarate, teriflunomide], or any second-line therapy [fingolimod, siponimod, natalizumab, ocrelizumab, cladribine, ofatumumab, alemtuzumab] during follow-up), history of infectious mononucleosis (yes, no, or unknown), smoking status (current smoking or nonsmoking status at diagnosis), alcohol consumption (yes or no), World Health Organization BMI category calculated as weight in kilograms divided by height in meters squared (underweight, <18.5; normal, 18.5-24.9; overweight, 25.0-29.9; and obesity, ≥30), physical activity, and sun exposure habits. Physical activity at diagnosis was categorized as low (physical activity without sweating for <2 hours per week), moderate (physical activity without sweating for ≥2 hours per week), moderate to high (regularly exercising with sweating for ≥30 minutes 1 to 2 times per week, or high (regularly exercising with sweating for ≥30 minutes at least 3 times per week). Based on 3 questions regarding sun exposure at diagnosis (sunbathing in Sweden, traveling to sunnier countries, and use of sunbeds), in which each answer alternative was given a number ranging from 1 (the lowest exposure) to 4 (the highest exposure), we constructed an index by adding the numbers together and thus acquired a value between 3 and 12. Sun exposure was then dichotomized based on the median value into high (≥6) or low (<6) exposure.

We performed several supplemental analyses. To assess potential collinearity among variables, Spearman correlation coefficients were calculated between educational level and both lifestyle factors and treatment. Additionally, variance inflation factors (VIFs) were examined in regression models. Treatment was further adjusted for by using the proportion of the follow-up time that patients received first-line treatment or second-line treatment. In a sensitivity analysis, we excluded participants for whom the self-reported educational level was higher than the registered educational level (n = 540, 14.5%). The implications of educational level for disease outcomes were analyzed and limited to participants with Nordic ancestry (participants born in Sweden, Norway, Finland, Denmark, or Iceland, with parents who had not immigrated from outside the Nordic countries). Subanalysis restricted to participants with Nordic ancestry to reduce genetic heterogeneity, as well as potential differences in educational level and other exposures. The impact of educational level 5 years prior to disease onset date was analyzed to reveal potential implications of reverse causation. We also performed a sensitivity analysis in which data from the EIMS and GEMS studies were analyzed separately, as well as a sensitivity analysis limited to patients with a disease onset date within 2005 to 2019 when second-line treatments had become available. We performed separate analyses limited to participants who received only first-line treatment or only second-line treatment during follow-up. Finally, to explore whether the observed association between educational level and MS progression was mediated by lifestyle factors and treatment, we conducted a mediation analysis.^18^ Educational level was treated as the independent variable, with CDW as the outcome. Treatment and lifestyle factors were assessed as potential mediators. Separate models were run to estimate the proportion of the effect of education level explained by treatment and lifestyle factors, both individually and in combination. Statistical significance was ascertained using a 2-sided threshold of *P* < .05. All analyses were conducted using SAS, version 9.4 (SAS Institute Inc).

## Results

Our study included 3695 participants with MS, of whom 2656 (71.9%) were female and 1039 (28.1%) were male. Mean (SD) age at diagnosis was 39.1 (9.1) years. There were differences in characteristics across groups with varying educational levels. Participants with postsecondary education were younger at diagnosis and had a higher proportion of females compared with those with lower levels of education. Patients with postsecondary education also reported a higher prevalence of past infectious mononucleosis, a higher level of sun exposure, lower BMI, and higher levels of physical activity. Additionally, they were less likely to smoke and more likely to consume alcohol. Detailed characteristics of participants by educational level are presented in Table 1.

**Table 1.** Characteristics of Participants With Multiple Sclerosis Overall and by Educational Level

Characteristic	Participants, No. (%)				*P* value
	Total (n = 3695)	Educational level			
		Postsecondary (n = 1607)	Secondary (n = 1644)	Presecondary (n = 444)	
Follow-up, mean (SD), y	10.4 (5.4)	10.5 (5.4)	10.6 (5.3)	8.9 (5.7)	<.001
Age at disease onset, mean (SD), y	37.2 (8.8)	36.0 (8.4)	37.1 (8.3)	42.2 (10.2)	<.001
Age at diagnosis, mean (SD), y	39.1 (9.1)	37.9 (8.8)	39.1 (8.7)	43.9 (10.4)	<.001
Sex					
Female	2656 (71.9)	1225 (76.2)	1150 (70.0)	281 (63.3)	<.001
Male	1039 (28.1)	382 (23.8)	494 (30.0)	163 (36.7)	
Nordic origin	3142 (85.0)	1329 (82.7)	1418 (86.3)	395 (89.0)	.001
Baseline EDSS score, mean (SD)	2.0 (1.7)	1.7 (1.5)	2.1 (1.7)	2.7 (2.0)	<.001
MSIS-29 physical subscale score, mean (SD)	23.2 (22.4)	19.5 (20.6)	25.7 (23.2)	28.9 (23.7)	<.001
MSIS-29 psychological subscale score, mean (SD)	30.7 (23.8)	28.3 (23.1)	32.3 (24.3)	35.0 (23.6)	<.001
Baseline SDMT score, mean (SD)	50.6 (12.1)	53.4 (11.6)	48.3 (12.1)	48.1 (11.8)	<.001
No treatment	180 (4.9)	65 (4.0)	67 (4.1)	48 (10.8)	<.001
First-line treatment^a^	1662 (45.0)	673 (41.9)	757 (46.1)	232 (52.3)	
Second-line treatment^b^	1853 (50.2)	869 (54.1)	820 (49.9)	164 (36.9)	
Time from diagnosis to treatment initiation, y^c^	0.7 (2.3)	0.7 (2.2)	0.7 (2.3)	0.8 (2.5)	.16
Proportion of follow-up time receiving treatment mean (SD)^c^	0.7 (0.3)	0.8 (0.3)	0.7 (0.3)	0.6 (0.4)	<.001
Visits per year, mean (SD), No.	1.1 (1.0)	1.1 (1.0)	1.0 (0.8)	1.2 (1.5)	<.001
History of infectious mononucleosis	626 (17.1)	326 (20.4)	249 (15.3)	51 (11.6)	<.001
Current smoking status	988 (27)	343 (21.3)	492 (29.9)	153 (34.5)	<.001
Alcohol consumption	2274 (61.5)	1054 (65.6)	988 (60.1)	232 (52.3)	<.001
Low sun exposure	1483 (40.1)	587 (36.5)	698 (42.5)	198 (44.6)	<.001
BMI^d^					
Underweight	103 (2.8)	43 (2.7),	38 (2.3)	22 (5.0)	<.001
Normal weight	1970 (53.3)	996 (62.0)	778 (47.3)	196 (44.1)	
Overweight	1104 (29.9)	404 (25.1)	556 (33.8)	144 (32.4)	
Obesity	518 (14.0)	164 (10.2)	272 (16.6)	82 (18.5)	
Low physical activity	642 (17.8)	223 (14.1)	325 (20.2)	94 (22.2)	<.001
Moderate activity	1528 (42.3)	621 (39.4)	709 (44.0)	198 (46.7)	
Moderate to high or high activity level	1441 (39.9)	733 (46.5)	576 (35.8)	132 (31.1)	

Abbreviations: BMI, body mass index (calculated as weight in kilograms divided by height in meters squared); EDSS, Expanded Disability Status Scale; MSIS-29, Multiple Sclerosis Impact Scale; SDMT, Symbol Digit Modalities Test.

^a^ Only first-line treatment during follow-up.

^b^ Second-line treatment during follow-up regardless of whether they had first-line treatment.

^c^ Treated with disease-modifying therapies.

^d^ BMI categories as defined by the World Health Organization were underweight, <18.5; normal, 18.5-24.9; overweight, 25.0-29.9; and obesity, ≥30.

The association of educational level with lifestyle factors and treatment type was weak to moderate, with Spearman correlation coefficients ranging from −0.15 to 0.12, indicating a low risk of multicollinearity. This finding was further supported by the VIF assessments, which remained below conventional thresholds (VIF <2), suggesting that multicollinearity was unlikely to alter the results.

At baseline, participants with presecondary education had higher EDSS and MSIS-29 scores and lower SDMT scores compared with those with higher educational levels (Table 1). Despite these worse clinical profiles, participants with presecondary education were less frequently treated with second-line therapies. The presecondary education group had the largest proportion of patients not receiving treatment at baseline and during follow-up. Among patients receiving treatment, no significant difference was observed between educational groups in time to treatment initiation, but the proportion of follow-up time spent receiving treatment was lower among those with presecondary education (Table 1). When excluding patients with disease onset before 2005, disparities in treatment-related factors persisted. The proportion of the patients receiving first- and second-line treatment, by year of disease onset (in 5-year intervals) and educational level, is provided in eTable 1 in Supplement 1.

### Specific Disability Outcomes

Compared with a postsecondary educational level, presecondary educational level was associated with an increased risk of CDW in the unadjusted analysis (HR, 1.37; 95% CI, 1.18-1.58), but this association was attenuated and was nonsignificant after adjustment for lifestyle factors and treatment (adjusted hazard ratio [AHR], 1.14; 95% CI, 0.97-1.33) (Table 2). Similarly, an association between presecondary educational level and risk of patient-reported physical worsening was observed in the unadjusted analysis (HR, 1.32; 95% CI, 1.06-1.65) but was attenuated and nonsignificant in the fully adjusted model (AHR, 1.14; 95% CI, 0.90-1.44). We observed no associations between presecondary educational level and the risk of either worsening on the MSIS-29 psychological subscale (AHR, 1.00; 95% CI, 0.79-1.26) or cognitive worsening measured by SDMT performance over the 15-year follow-up (AHR, 1.05; 95% CI, 0.76-1.46).

**Table 2.** Unfavorable Outcomes Among Patients With MS by Educational Level

Educational level	Patients No./total No. (%)	Time to outcome, including disability worsening, mean (SD), y	Unadjusted model, HR (95% CI)^a^	Partially adjusted model, HR (95% CI)^b^	Fully adjusted model, HR (95% CI)^c^
**Clinical disease worsening**					
Postsecondary	816/1607 (50.8)	6.9 (4.9)	1 [Reference]	1 [Reference]	1 [Reference]
Secondary	905/1644 (55.1)	6.6 (4.8)	1.12 (1.02-1.23)	1.08 (0.98-1.19)	1.06 (0.96-1.17)
Presecondary	247/444 (55.6)	5.6 (4.4)	1.37 (1.18-1.58)	1.19 (1.02-1.39)	1.14 (0.97-1.33)
**Physical worsening**					
Postsecondary	463/1170 (39.6)	5.2 (4.0)	1 [Reference]	1 [Reference]	1 [Reference]
Secondary	524/1151 (45.5)	5.3 (5.3)	1.17 (1.04-1.33)	1.15 (1.01-1.30)	1.08 (0.94-1.23)
Presecondary	95/228 (41.7)	4.1 (3.9)	1.32 (1.06-1.65)	1.24 (0.98-1.55)	1.14 (0.90-1.44)
**Psychological worsening**					
Postsecondary	587/1165 (50.4)	4.8 (4.2)	1 [Reference]	1 [Reference]	1 [Reference]
Secondary	601/1150 (52.3)	5.0 (5.1)	1.04 (0.92-1.16)	1.04 (0.92-1.18)	0.98 (0.87-1.11)
Presecondary	99/228 (43.4)	3.9 (4.4)	1.06 (0.85-1.32)	1.06 (0.86-1.33)	1.00 (0.79-1.26)
**Cognitive worsening**					
Postsecondary	310/1182 (26.2)	5.6 (3.4)	1 [Reference]	1 [Reference]	1 [Reference]
Secondary	280/1197 (23.4)	5.7 (3.5)	0.87 (0.74-1.03)	0.97 (0.82-1.15)	0.99 (0.83-1.17)
Presecondary	51/237 (21.5)	4.7 (3.3)	0.96 (0.70-1.31)	1.01 (0.74-1.37)	1.05 (0.76-1.46)

Abbreviation: HR, hazard ratio.

^a^ Unadjusted Cox model.

^b^ Adjusted for age at diagnosis, sex, past infectious mononucleosis, smoking status, alcohol consumption, sun exposure, body mass index, and physical activity.

^c^ Adjusted for age at diagnosis, sex, past infectious mononucleosis, smoking status, alcohol consumption, sun exposure, body mass index, physical activity, and treatment.

### Analysis of Longitudinal EDSS Changes

The unadjusted analysis found that patients with a presecondary educational level have faster disability worsening measured by the EDSS (β, 0.03; 95% CI, 0.01-0.04) (Table 3 and Figure). However, after adjusting for lifestyle factors and treatment-related factors, no association was found between educational level and changes in the EDSS over the 15-year follow-up period (β, 0.001 [95% CI, −0.01 to 0.01] for participants with secondary education, and β, 0.001 [95% CI, −0.02 to 0.02] for those with presecondary education, compared with those with postsecondary education). No long-term associations were observed between educational level and either patient-reported physical or psychological worsening, nor were there any significant changes in SDMT scores (eTables 2-4, eFigures 1-3 in Supplement 1).

**Table 3.** Annual Change in Expanded Disability Status Scale Scores Over 15 Years After Multiple Sclerosis Diagnosis for Patients With Different Educational Levels

	Basic mixed-effects model^a^		Fully adjusted model^b^	
	β (95% CI)	*P* value	β (95% CI)	*P* value
**Educational level**				
Postsecondary	1 [Reference]	NA	1 [Reference]	NA
Secondary	0.413 (0.304 to 0.522)	<.001	0.299 (0.192 to 0.406)	<.001
Presecondary	0.857 (0.676 to 1.038)	<.001	0.576 (0.395 to 0.757)	<.001
**Time × education level**				
Postsecondary	1 [Reference]	NA	1 [Reference]	NA
Secondary	0.005 (−0.005 to 0.015)	.31	0.001 (−0.009 to 0.011)	.84
Presecondary	0.026 (0.008 to 0.044)	.004	0.001 (−0.017 to 0.019)	.91

Abbreviation: NA, not applicable.

^a^ The basic mixed-effects model included educational level, time, and their interaction.

^b^ The fully adjusted model was further adjusted for age at diagnosis, sex, past infectious mononucleosis, smoking status, alcohol consumption, sun exposure, body mass index, physical activity level, and treatment type.

**Figure.**
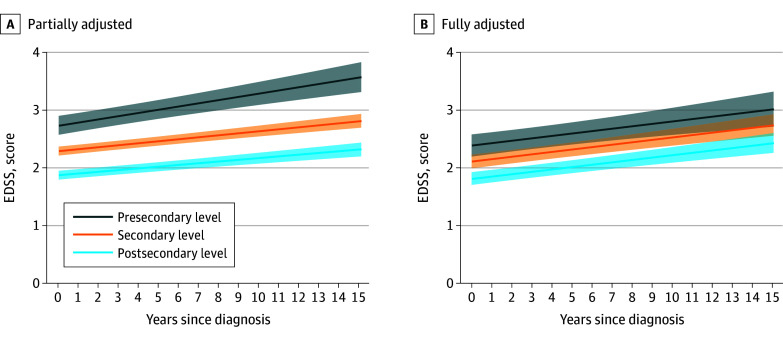
Trajectories of Expanded Disability Status Scale (EDSS) Scores During 15-Year Follow-Up Across Educational Levels Among Participants With Multiple Sclerosis A, Adjusted for educational level, time since baseline (ie, date of multiple sclerosis diagnosis), and their interaction. B, Further adjusted for age at diagnosis, sex, past infectious mononucleosis, smoking status, alcohol consumption, sun exposure, body mass index, physical activity, and treatment type. The figure represents marginal effects of educational levels on trajectories of EDSS scores. The shaded areas represent the 95% CIs of the estimated mean EDSS scores.

### Supplemental Analyses

Our findings were robust across several supplemental analyses. The results remained consistent when the analysis was limited to participants for whom self-reported and registered educational levels were concordant, as well as when restricted to participants of Nordic ancestry (adjusted β of EDSS progression, 0.01 [95% CI, −0.01 to 0.02] for participants with a presecondary educational level compared with a postsecondary educational level). The findings were consistent when we analyzed the EIMS and GEMS cohorts separately, with no associations found between educational level and outcomes in the adjusted models (β of EDSS progression, −0.02 [95% CI, −0.05 to 0.02] for those with presecondary educational level in the EIMS study and β, 0.00 [95% CI, −0.02 to 0.02] for the corresponding level in the GEMS study). No association between presecondary educational level and EDSS-related disability progression was observed when we excluded participants with disease onset before 2005 when second-line treatments became available (adjusted β, −0.01, 95% CI, −0.04 to 0.01). Additionally, after adjustment for clinical and lifestyle-related variables, no association was observed between presecondary educational level and risk of CDW among participants who received only first-line treatment during follow-up (HR, 1.06; 95% CI, 0.86-1.31) or among those who received second-line treatment (HR, 1.17; 95% CI, 0.93-1.54).

In mediation analysis, we found that treatment and lifestyle factors together explained 79.7% of the association between presecondary educational level and increased risk of CDW. Treatment alone accounted for 29.1% of the association, while lifestyle factors (including smoking, alcohol consumption, BMI, physical activity, and sun exposure) accounted for an additional 50.6%. When analyzed separately, treatment and physical activity explained the largest proportion of the association, whereas smoking and alcohol consumption had a more modest impact. When models were adjusted for all factors simultaneously, no association remained between educational level and MS progression (AHR, 1.14; 95% CI, 0.97-1.33).

## Discussion

In this large, population-based study, we investigated the association between educational level and MS progression, with a focus on potential confounding and mediation by lifestyle factors and treatment. Results of the unadjusted analysis suggested that presecondary educational level was associated with faster disability progression. However, after adjusting for lifestyle factors and treatment, these associations were attenuated and no longer significant, indicating that educational level itself does not appear to be directly associated with disability progression in MS.

Our mediation analysis revealed that treatment choices and lifestyle factors together may explain approximately 80% of the observed association between educational level and disability progression. Treatment choices accounted for nearly one-third of the observed association, while lifestyle factors collectively explained more than half of the mediation effect. These findings suggest that educational level indirectly mediates the association between educational level and disability worsening in MS, by shaping health behaviors and treatment patterns rather than exerting a direct effect on disease progression.

Although access to health care was similar across educational groups as reflected by comparable time from symptom onset to diagnosis and higher health care use among individuals with a presecondary educational level, treatment disparities persisted. Patients with a presecondary educational level were less likely to initiate second-line therapy and had a shorter cumulative treatment time. Given the increasing evidence supporting early, aggressive treatment strategies in MS, this disparity may contribute to worse outcomes among individuals with lower socioeconomic status.

Additionally, individuals with a presecondary education level had a higher prevalence of lifestyle factors associated with disease progression, such as smoking status and a lower level of physical activity. In the mediation analysis, lifestyle factors played a larger role than treatment in the association between presecondary educational level and increased risk of CDW, reinforcing the importance of addressing modifiable risk factors in MS care.

Given that several common lifestyle risk factors for disease progression are associated with educational level, the risk of MS-related disability progression, cognitive decline, and health related quality of life decline may differ between educational groups depending on the prevalence and distribution of these risk factors, which can vary across geographical areas and time periods. The relationship between educational level and outcomes can also differ across countries due to differences in health care systems, social support structures, and cultural factors. In Sweden, the universal health care system provides equitable access to medical services, likely mitigating some of the disparities that might be more pronounced in countries with less comprehensive coverage. However, educational level may be associated with other unmeasured factors, such as health literacy, stress, or social support, which could alter disease outcomes differently in other contexts. Therefore, caution should be exercised when generalizing these findings to populations outside of Sweden.

### Strengths and Limitations

Key strengths of our study include its large sample size, population-based design, and comprehensive data on clinical, demographic, and lifestyle factors. Additionally, a comparison of key demographic and clinical characteristics between the study population and the full national registry showed similar distributions in sex, age at onset, disease duration, and treatment patterns, supporting the generalizability of our findings within the context of the Swedish population. The validation of self-reported educational level against Swedish registry data further strengthens the reliability of our exposure measure.

We acknowledge several study limitations. The EDSS is an ordinal and nonlinear scale and linear mixed-effects models are not ideally suited for analyzing such outcomes. In this study, these models were used only to illustrate group-level EDSS trajectories over time. The reliance on self-reported data for lifestyle risk factors introduces the potential for recall bias, particularly in a study in which data on lifestyle habits were collected retrospectively. Despite this limitation, similar results were observed when the EIMS and GEMS cohorts were analyzed separately, suggesting that recall bias did not substantially affect our findings. Selection bias was minimized by the population-based design, which ensured broad representation across different educational levels. Additionally, the proportions of participants with various educational levels in our study were in accordance with those in the general Swedish population,^19^ suggesting that the findings are likely not substantially impacted by selection bias related to educational level.

## Conclusions

In this cohort study, the observed association between educational level and MS progression was largely explained by differences in treatment patterns and modifiable lifestyle risk factors, suggesting that education level itself may not be independently associated with disability progression in MS. These results emphasize the importance of addressing health behaviors and ensuring equitable access to effective treatment across educational groups.

## Notes

Supplement 1. **eTable 1.** Proportion of participants on low/moderate- and high-efficacy disease-modifying treatment during the follow-up period, by educational attainment **eTable 2.** β coefficient and 95% CI of MSIS-29 physical score over 15 years after diagnosis of MS across educational levels **eTable 3.** β coefficient and 95% CI of MSIS-29 psychological score over 15 years after diagnosis of MS across educational levels **eTable 4.** β coefficient and 95% CI of EDSS score over 15 years after diagnosis of MS across educational levels **eFigure 1.** Trajectories of MSIS-29 physical component during 15-year follow-up across educational levels among MS participants **eFigure 2.** Trajectories of MSIS-29 psychological component during 15-year follow-up across educational levels among MS participants **eFigure 3.** Trajectories of SDMT during 15-year follow-up across educational levels among MS participants

Supplement 2.
Data Sharing Statement
