# Supplementary material for: Education, Lifestyle Risk Factors, and Treatment Choices and Multiple Sclerosis Progression
Source: JAMA Netw Open. 2025 Jul 11;8(7):e2520142. doi: 10.1001/jamanetworkopen.2025.20142 (PMC12254889; doi:10.1001/jamanetworkopen.2025.20142)
Supplement: Supplement 1. — eTable 1. Proportion of participants on low/moderate- and high-efficacy disease-modifying treatment during the follow-up period, by educational attainment eTable 2. β coefficient and 95% CI of MSIS-29 physical score over 15 years after diagnosis of MS across educational levels eTable 3. β coefficient and 95% CI of MSIS-29 psychological score over 15 years after diagnosis of MS across educational levels eTable 4. β coefficient and 95% CI of EDSS score over 15 years after diagnosis of MS across educational levels eFigure 1. Trajectories of MSIS-29 physical component during 15-year follow-up across educational levels among MS participants eFigure 2. Trajectories of MSIS-29 psychological component during 15-year follow-up across educational levels among MS participants eFigure 3. Trajectories of SDMT during 15-year follow-up across educational levels among MS participants [file jamanetwopen-e2520142-s001.pdf]

Supplemental Online Content

Guo J, Olsson T, Hillert J, Alfredsson L, Hedström AK. Education, Lifestyle, and Treatment Choices and Multiple Sclerosis Progression.
JAMA Netw Open. 2025; 8(7): e2520142. doi:10.1001/jamanetworkopen.2025.20142

eTable 1. Proportion of participants on low/moderate- and high-efficacy disease-modifying treatment during the follow-up period, by educational attainment.

eTable 2. β coefficient and 95% CI of MSIS-29 physical score over 15 years after diagnosis of MS across educational levels.

eTable 3. β coefficient and 95% CI of MSIS-29 psychological score over 15 years after diagnosis of MS across educational levels.

eTable 4. β coefficient and 95% CI of EDSS score over 15 years after diagnosis of MS across educational levels.

eFigure 1. Trajectories of MSIS-29 physical component during 15-year follow-up across educational levels among MS participants.

eFigure 2. Trajectories of MSIS-29 psychological component during 15-year follow-up across educational levels among MS participants.

eFigure 3. Trajectories of SDMT during 15-year follow-up across educational levels among MS participants.

This supplemental material has been provided by the authors to give readers additional information about their work.

© 2025 Guo J. et al. *JAMA Network Open*.

eTable 1. Proportion of participants on low/moderate- and high-efficacy disease-modifying treatment during the follow-up period, by educational attainment.

Post-secondary education						
Year of disease onset	N	Follow-up years	Time on treatment[1]	No treatment (%)	Low-efficacy[2] (%)	High-efficacy[3] (%)
1995-1999	283	11.7 (5.7)	0.8 (0.3)	8.1	51.9	39.9
2000-2004	392	9.1 (4.6)	0.9 (0.3)	4.6	49.7	45.7
2005-2009	371	7.5 (3.5)	0.9 (0.2)	3.2	36.7	60.1
2010-2014	353	6.2 (2.1)	0.9 (0.2)	2.3	35.7	62.0
Secondary education						
Year of disease onset	N	Follow-up years	Time on treatment[1]	No treatment (%)	Low-efficacy[2] (%)	High-efficacy[3] (%)
1995-1999	334	11.7 (5.8)	0.8 (0.3)	7.8	54.8	37.4
2000-2004	435	9.3 (4.4)	0.8 (0.3)	5.8	49.0	45.3
2005-2009	426	7.6 (3.6)	0.9 (0.2)	1.9	47.4	50.7
2010-2014	305	5.8 (2.4)	0.9 (0.2)	1.9	34.8	63.3
Pre-secondary education						
Year of disease onset	N	Follow-up years	Time on treatment[1]	No treatment (%)	Low-efficacy[2] (%)	High-efficacy[3] (%)
1995-1999	128	12.3 (5.8)	0.7 (0.4)	14.8	68.8	16.4
2000-2004	115	10.2 (4.1)	0.8 (0.4)	10.4	65.2	24.4
2005-2009	60	8.0 (3.5)	0.8 (0.3)	13.3	41.7	45.0
2010-2014	60	6.0 (2.2)	0.9 (0.3)	10.0	35.0	55.0

[1]proportion of the follow-up time on treatment; [2]only low-efficacy treatment during follow-up; [3]high-efficacy treatment during follow-up regardless of if they have had low-efficacy treatment.

© 2025 Guo J. et al. *JAMA Network Open*.

eTable 2. β coefficient and 95% CI of MSIS-29 physical score over 15 years after diagnosis of MS across educational levels.

Effect	Basic model		Multi-adjusted model	
	β (95% CI)	P value	β (95% CI)	P value
Educational level				
Post-secondary school	Reference		Reference	
Secondary school	6.64 (4.88, 8.41)	<0.0001	4.94 (3.19, 6.69)	<0.0001
Pre-secondary school	10.78 (7.6, 13.96)	<0.0001	8.5 (5.32, 11.69))	<0.0001
Time*education level				
Post-secondary school	Reference		Reference	
Secondary school	-0.05 (-0.22, 0.12)	0.56	-0.03 (-0.61, 0.13)	0.77
Pre-secondary school	-0.15 (-0.51, 0.21)	0.40	-0.24 (-0.20, 0.15)	0.20

The basic model included education level, time, and their interaction term. The multi-adjusted model was further adjusted for age at diagnosis, sex, past IM, smoking, alcohol consumption, sun exposure, BMI, physical activity, and treatment.

© 2025 Guo J. et al. *JAMA Network Open*.

eTable 3. β coefficient and 95% CI of MSIS-29 psychological score over 15 years after diagnosis of MS across educational levels.

Effect	Basic model		Multi-adjusted model	
	β (95% CI)	P value	β (95% CI)	P value
Educational level				
Post-secondary school	Reference		Reference	
Secondary school	4.94 (2.99, 6.89)	<0.0001	3.94 (1.98, 5.90)	<0.0001
Pre-secondary school	9.16 (5.67, 12.65)	<0.0001	7.94 (4.37, 11.5)	<0.0001
Time*education level				
Post-secondary school	Reference		Reference	
Secondary school	-0.09 (-0.27, 0.10)	0.36	-0.08 (-0.64, 0.19)	0.39
Pre-secondary school	-0.16 (-0.57, 0.24)	0.42	-0.22 (-0.28, 0.11)	0.30

The basic model included education level, time, and their interaction term. The multi-adjusted model was further adjusted for age at diagnosis, sex, past IM, smoking, alcohol consumption, sun exposure, BMI, physical activity, and treatment.

© 2025 Guo J. et al. *JAMA Network Open*.

eTable 4. β coefficient and 95% CI of EDSS score over 15 years after diagnosis of MS across educational levels.

Effect	Basic model		Multi-adjusted model	
	β (95% CI)	P value	β (95% CI)	P value
Educational level				
Post-secondary school	Reference		Reference	
Secondary school	-5.24 (-6.41, -4.07)	<0.0001	-4.12 (-5.28, -2.95)	<0.0001
Pre-secondary school	-6.68 (-8.78, -4.57)	<0.0001	-5.12 (-7.25, -2.99)	<0.0001
Time*education level				
Post-secondary school	Reference		Reference	
Secondary school	0.07 (-0.04, 0.19)	0.22	0.08 (-0.15, 0.35)	0.19
Pre-secondary school	0.00 (-0.25, 0.24)	0.98	0.10 (-0.04, 0.20)	0.42

The basic model included education level, time, and their interaction term. The multi-adjusted model was further adjusted for age at diagnosis, sex, past IM, smoking, alcohol consumption, sun exposure, BMI, physical activity, and treatment.

© 2025 Guo J. et al. *JAMA Network Open*.

eFigure 1. Trajectories of MSIS-29 physical component during 15-year follow-up across educational levels among MS participants.

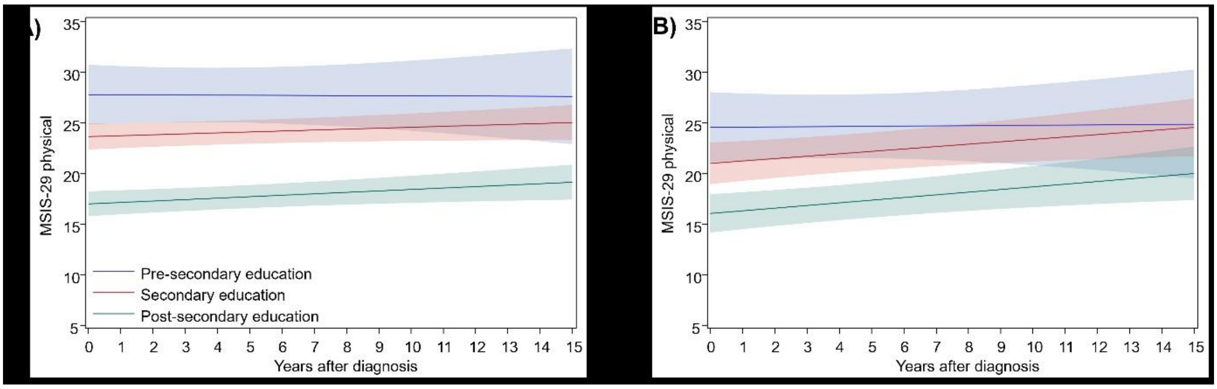

The figure represents marginal effects of educational level on trajectories of MSIS-29 physical component. The bands represent the 95% CI of estimated mean MSIS-29 physical component. In the crude model (A), we adjusted for education level, time, and their interaction term. The multi-adjusted model (B) was adjusted for age at diagnosis, sex, past IM, smoking, alcohol consumption, sun exposure, BMI, physical activity, and treatment.

© 2025 Guo J. et al. *JAMA Network Open*.

eFigure 2. Trajectories of MSIS-29 psychological component during 15-year follow-up across educational levels among MS participants.

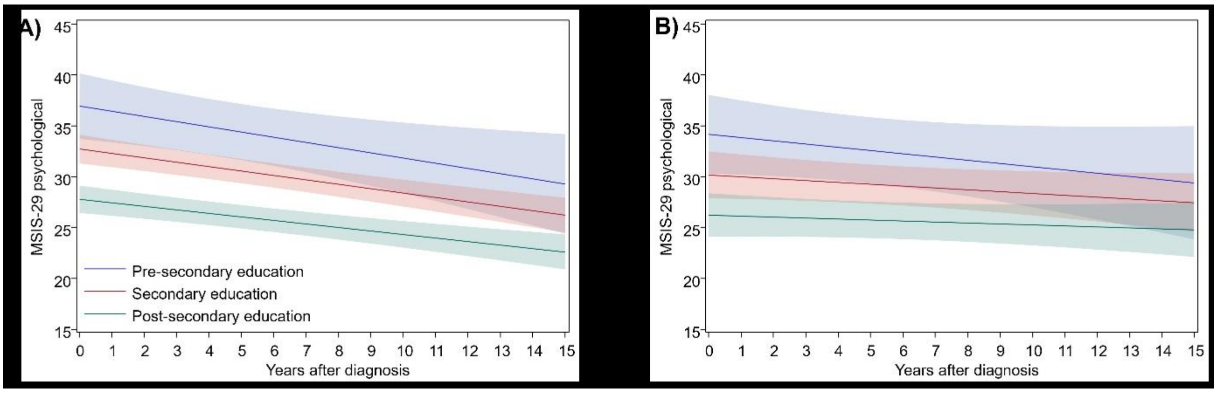

The figure represents marginal effects of educational level on trajectories of MSIS-29 psychological component. The bands represent the 95% CI of estimated mean MSIS-29 psychological component. In the crude model (A), we adjusted for education level, time, and their interaction term. The multi-adjusted model (B) was adjusted for age at diagnosis, sex, past IM, smoking, alcohol consumption, sun exposure, BMI, physical activity, and treatment.

© 2025 Guo J. et al. *JAMA Network Open*.

eFigure 3. Trajectories of SDMT during 15-year follow-up across educational levels among MS participants.

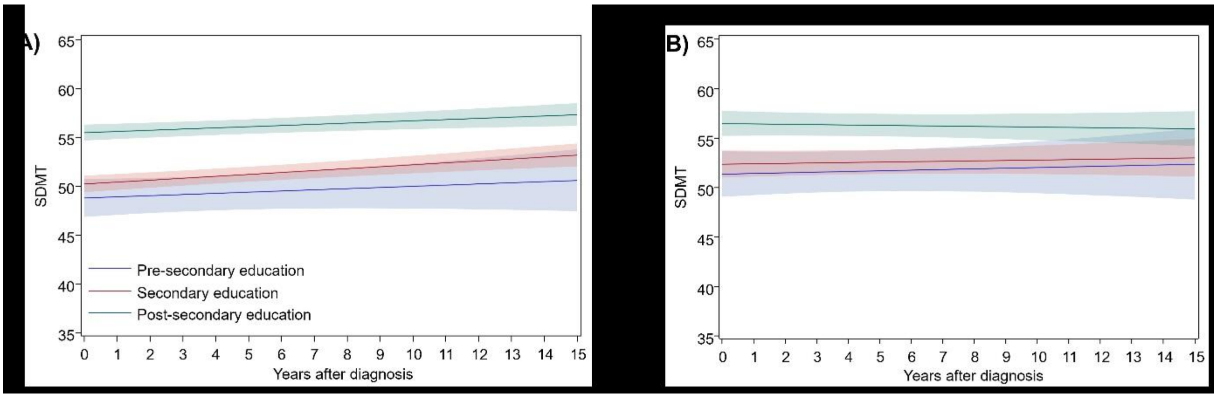

The figure represents marginal effects of educational level on trajectories of SDMT. The bands represent the 95% CI of estimated mean SDMT. In the crude model (A), we adjusted for education level, time, and their interaction term. The multi-adjusted model (B) was adjusted for age at diagnosis, sex, past IM, smoking, alcohol consumption, sun exposure, BMI, physical activity, and treatment.

© 2025 Guo J. et al. *JAMA Network Open*.
