# Supplementary material for: Education, Lifestyle Risk Factors, and Treatment Choices and Multiple Sclerosis Progression
Source: JAMA Netw Open. 2025 Jul 11;8(7):e2520142. doi: 10.1001/jamanetworkopen.2025.20142 (PMC12254889; doi:10.1001/jamanetworkopen.2025.20142)
Supplement: Supplement 2. — Data Sharing Statement [file jamanetwopen-e2520142-s002.pdf]

Data Sharing Statement

Guo. Education, Lifestyle Risk Factors, and Treatment Choices and Multiple Sclerosis Progression. *JAMA Netw Open*. Published July 11, 2025.
doi:10.1001/jamanetworkopen.2025.20142

Data
Data available: No

Additional Information
Explanation for why data not available: Anonymized data underlying this article will be shared on reasonable request from any qualified investigator that wants to analyze questions that are related to the published article.
